# Massive transfusion triggers in severe trauma: Scoping review*

**DOI:** 10.1590/1518-8345.2574.3102

**Published:** 2018-11-29

**Authors:** Cristina Estebaranz-Santamaría, Ana María Palmar-Santos, Azucena Pedraz-Marcos

**Affiliations:** 1 Hospital Universitario La Paz, Madrid, Espanha.; 2 Universidad Autónoma de Madrid, Madrid, Espanha.

**Keywords:** Blood Coagulation Disorders, Wounds and Injuries, Blood Transfusion, Resuscitation, Emergency Medical Service

## Abstract

**Objective:**

to identify the predictive variables or the massive transfusion triggers in severely traumatized patients through the existing scales.

**Method:**

a review of the literature was carried out using the Scoping Review method across the electronic databases CINAHL, MEDLINE, LILACS, the Cochrane and IBECS libraries, and the Google Scholar search tool.

**Results:**

in total, 578 articles were identified in the search and the 36 articles published in the last ten years were included, of which 29 were original articles and 7 review articles. From the analysis, scales for massive transfusion and their predictive triggers were examined.

**Conclusion:**

the absence of universal criteria regarding the massive transfusion triggers in traumatized patients has led to the development of different scales, and the studies on their validation are considered relevant for the studies about when to initiate this strategy.

## Introduction

Hemorrhage is the leading cause of potentially preventable death among trauma patients, and early intervention within the first 24 hours after the event takes place is critical in terms of survival^(^ ^1^ ^-^ ^2^ ^)^. In this way, trauma injuries have become a public health problem, which may have an impact not only on mortality, but also on years of life lost in younger adults^(^ ^3^ ^)^.

Gradually, in the last decades, new strategies and protocols have been developed with the aim of preventing the so-called “lethal triad”, with its components: acidosis, hypothermia and coagulopathy, caused by the great loss of blood^(^ ^4^ ^-^ ^5^ ^)^. In this context, aiming at its prevention and resolution, the Damage Control Surgery (DCS) emerged, which is exclusively used in the operating room and, over the years, it has evolved towards the concept of Damage Control Resuscitation (DCR), encompassing the out-of-hospital and hospital emergency areas^(^ ^2^ ^,^ ^6^ ^-^ ^7^ ^)^. Within the main strategies of the DCR, it is worth highlighting the so-called Massive Transfusion (MT), which consists of the administration of ten or more blood products (red blood cells, plasma and platelets) within the first 24 hours, according to the traditional concept^(^ ^4^ ^,^ ^8^ ^-^ ^9^ ^)^. Other authoritative definitions include four or more components within the first hour^(^ ^10^ ^)^, or five or more components within the first four hours^(^ ^11^ ^-^ ^13^ ^)^.

The main advantage of the administration of MT in relation to the other strategies of the DCR, such as fluid therapy, is that it improves tissue oxygenation. For this reason, its early initiation is prioritized through the early transfer to a hospital center, although it is not a standardized procedure available in the out-of-hospital emergency itself^(^ ^9^ ^,^ ^14^ ^)^. Most importantly, MT has shown an increase in survival, a decrease in subsequent transfusion requirements and a decline in the average length of hospital stay^(^ ^8^ ^,^ ^14^ ^-^ ^16^ ^)^.

However, not all severe traumatized patients will be the receivers of this strategy, so predicting the real need for MT is considered essential, and it may only be performed after assessing several clinical, analytical and anatomical parameters, which are described as predictors or “triggers”^(^ ^9^ ^,^ ^17^ ^)^. For their measurement and interpretation, scales combining different types of variables have been developed in order to achieve a high predictive value and increase their specificity. However, despite the diversity of scales investigated and the frequent validation studies, a consensus on the “triggers” of MT has not yet been established^(^ ^7^ ^)^.

The objective of this study was to perform a scoping review to identify the clinical, physiological and anatomical predictive variables of massive transfusion, or triggers, in severely traumatized patients through the existing scales.

## Method

The theoretical framework used for the scoping review was proposed in 2005 by two English authors^(^ ^18^ ^)^. This methodology uses an approach aiming at a narrative synthesis, which is ideal for comparing scientific articles and contemplates the following steps that were considered in the present study: 1) identification of the research question or questions; 2) identification of relevant studies; 3) selection of studies; 4) data extraction; 5) synthesis and report of results; and 6) dissemination^(^ ^18^ ^-^ ^19^ ^)^.

Starting with the first phase of this methodology, the research question from which the scoping review began was: What are the predictive variables or the triggers for initiating massive transfusion scales in traumatized patients? For its resolution, a second phase characterized by the identification of relevant studies was initiated through a main search carried out over several months, according to previous recommendations from experts on the search terms and appropriate databases for its development.

**Figure 1:**
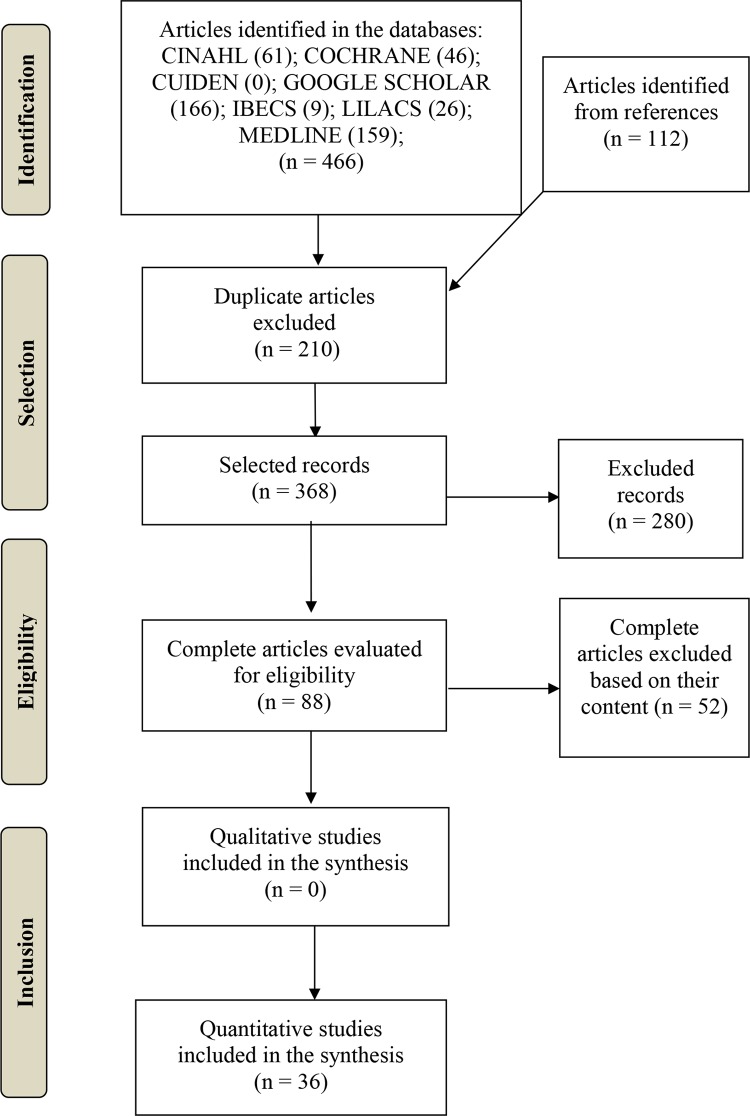
PRISMA flow diagram on the identification, selection and inclusion of articles. Madrid, Spain, 2017

Thus, a search across the literature was carried out using the following electronic resources and databases: Cumulative Index to Nursing and Allied Health Literature (CINAHL), Medical Literature Analysis and Retrieval System Online (MEDLINE), Latin American Literature and Caribbean Health Sciences Literature (LILACS), Spanish Bibliographic Index of Health Sciences (IBECS), Cochrane Library and Google Scholar search tool, as shown in Figure 1. In these databases, combinations of the following concepts were used as search strategy: “Massive”, “Transfusion”, “Trauma”, “Predict*” and the descriptors: “Wounds and Injuries”, “Blood Transfusion”, with the Boolean operators “and” and “or”. Furthermore, in the databases in which the mentioned words were available, it was specified that those words appearing in the title and/or abstract fields were in English, Spanish, Portuguese or French. Regarding the years interval analyzed, it was decided to encompass the last ten years, including the current year of 2017, due to the contemporaneity of the massive transfusion, its continuous scientific interest, and the magnitude of the significant contributions on the research theme. In addition, some studies prior to the aforementioned date, resulting from the search, were initially analyzed because they were considered relevant for the understanding and development of predictive scales for massive transfusion.

For the selection of articles, in the third phase of the scoping review, original and review studies were included, considering both the areas related to the creation of scales and their subsequent validations, and the more specific investigations in which the triggers are analyzed individually, as well as other general concepts of massive transfusion.

To ensure that this set did not present biased results, making it difficult to extrapolate the conclusions to a specific population group, it was also crucial to adopt exclusion criteria for the final selection of articles and their eligibility. Those articles in which the population was pediatric or with non-traumatic MT etiology were excluded, although these populations were also receivers of the strategy.

In this way, 578 articles in total were obtained using the search strategy, of which 36 finally met the inclusion criteria. The distribution of the articles identified in the databases and the search and selection processes are illustrated in the flow diagram of Figure 1.

From the selected publications, the data corresponding to the fourth phase were extracted by a two phases analysis, beginning firstly with those studies that gave rise to the scales, being identified: general data (date and place), type of publication with its corresponding design type (prospective or retrospective), characteristics of the sample (incidence of MT), statistical results - sensitivity (S), specificity (E), Area Under the Curve (AUC), Odds Ratio (OR), positive predictive value (PPV) and negative predictive value (NPV) - and main conclusions. Secondly, other articles that aimed to validate the scales with other samples, analyze specific triggers in a particular way and review other concepts about MT were examined.

Finally, the phase of gathering and synthesis of the results was carried out with the main objective of presenting an overview of all the material, through a thematic construction organized for its subsequent dissemination phase. In this way, in this scoping review, both the original articles and the review articles were included in order to provide a detailed understanding of the issue to be analyzed, that is, the predictive variables or the massive transfusion triggers in severe trauma.

## Results

Of the 36 studies identified based on their typology, 29 original and 7 review studies were selected, which were divided into two groups according to the methodology used for their development. Thus, the first group of results corresponds to the analysis of 19 of the 36 original studies on massive transfusion scales and their validation. In the second group are the 10 remaining studies, together with the 7 review articles, including the specific studies on the triggers and the general concepts about MT. Starting with the first group, the scales with their respective predictive clinical, physiological and anatomical variables, arranged chronologically according to their development and implementation, are presented below^(^ ^20^ ^-^ ^37^ ^)^:

- Shock Index (SI): Systolic Blood Pressure (SBP), HR (Heart Rate)^(^ ^20^ ^-^ ^21^ ^)^.
- Emergency Transfusion Score (ETS): SBP, Focused Abdominal Sonography for Trauma (FAST), type of trauma, age and injury mechanism^(^ ^22^ ^-^ ^23^ ^)^.
- Trauma Associated Severe Hemorrhage (TASH): SBP, HR, Hemoglobin (Hb), Base excess (BE), FAST and trauma type^(^ ^24^ ^-^ ^25^ ^)^.
- *Schreiber*: Hemoglobin (Hb), International Normalized Ratio (INR), trauma type and sex^(^ ^26^ ^)^.
- *McLaughlin*: SBP, HR, pH and Hematocrit (Hct)^(^ ^27^ ^)^.
- Assessment of Blood Consumption (ABC): SBP, HR, FAST, trauma type^(^ ^28^ ^-^ ^29^ ^)^
- *Larson*: SBP, HR, Hb y BE^(^ ^30^ ^)^.
- *Vandromme*: SBP, HR, Hb, INR and Lactate^(^ ^31^ ^)^.
- Prince of Wales Hospital (PWH/Rainer): SBP, HR, FAST, trauma type, Hb, BE and Glasgow Coma Scale (GCS)^(^ ^5^ ^,^ ^32^ ^)^.
- Cincinnati Individual Transfusion Trigger (CITT): SBP, T, Hb, BE, INR and FAST^(^ ^33^ ^)^.
- Massive Transfusion Score (MTS) and Revised Massive Transfusion Score (RMTS): SBP, HR, T, Hb, BE, INR, FAST and trauma type^(^ ^34^ ^-^ ^35^ ^)^.
- Traumatic Bleeding Severity Score (TBSS): SBP, Lactate, FAST, type and age^(^ ^36^ ^-^ ^37^ ^)^.

**Figure 2:**
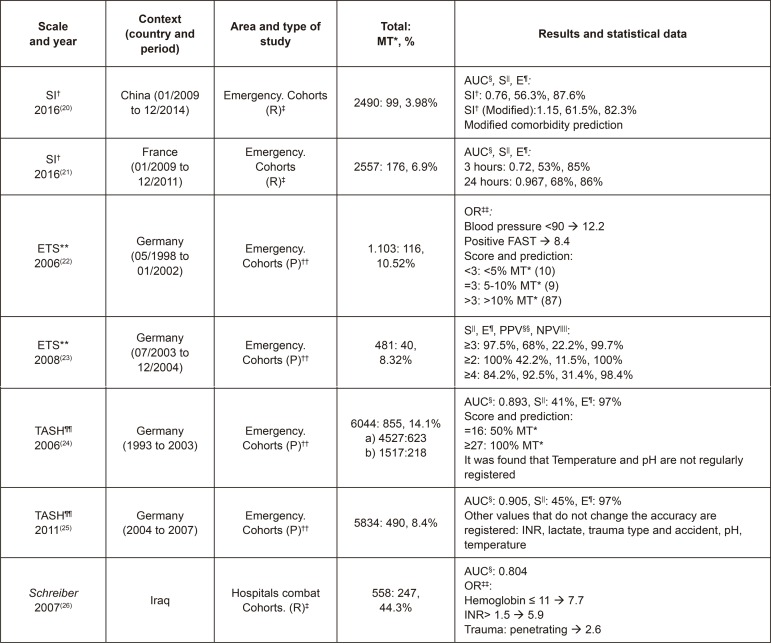
Characteristics of the studies on Shock Index (SI), Emergency Transfusion Score (ETS), Trauma Associated Severe Hemorrhage (TASH) and Schreiber. Madrid, Spain, 2017

^*^MT - Massive Transfusion, ^†^SI - Shock Index, ^‡^(R) - Retrospective, ^§^AUC - Area Under the Curve, ^||^S - Sensitivity, ^¶^E - Specificity, ^**^ETS - Emergency Transfusion Score, ^††^(P) - Prospective, ^‡‡^OR - Odds Ratio, ^§§^PPV - Positive Predictive Value, ^||||^NPV - Negative Predictive Value, ^¶¶^TASH: Trauma Associated Severe Hemorrhage

**Figure 3:**
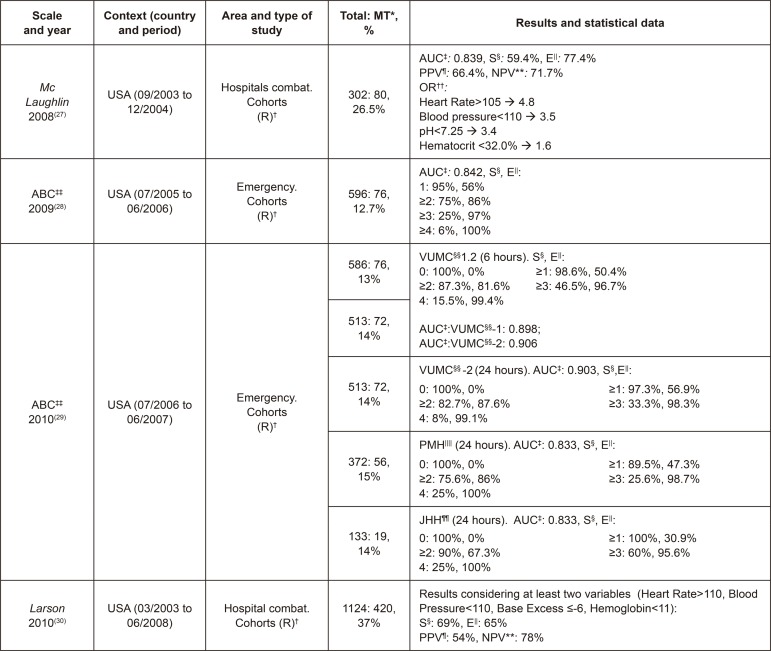
Characteristics of the studies on *McLaughlin*, Assessment of Blood Consumption (ABC) and *Larson*. Madrid, Spain, 2017

^*^MT - Massive Transfusion, ^†^(R) - Retrospective, ^‡^AUC - Area Under the Curve, ^§^S - Sensitivity, ^||^E - Specificity, ^¶^PPV - Positive Predictive Value, ^**^NPV - Negative Predictive Value, ^††^OR - Odds Ratio, ^‡‡^ABC - Assessment of Blood Consumption, ^§§^VUMC - Vanderbilt University Medical Center, ^||||^PMH - Parkland Memorial Hospital, ^¶¶^JHH - Johns Hopkins Hospital

**Figure 4:**
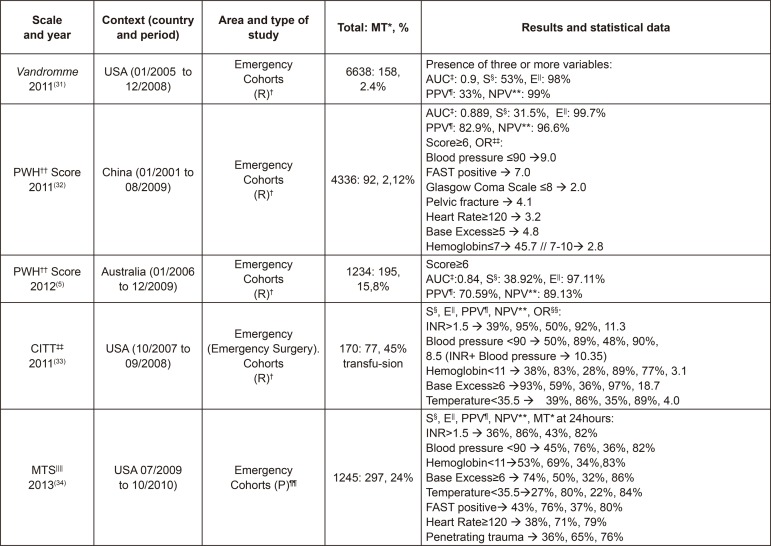
Characteristics of the studies on *Vandromme*, Prince of Wales Hospital (PWH) Score, Cincinnati Individual Transfusion Trigger Emergency (CITT) and Massive Transfusion Score (MTS). Madrid, Spain, 2017

^*^MT - Massive Transfusion, ^†^(R) - Retrospective, ^‡^AUC - Area Under the Curve, ^§^S - Sensitivity, ^||^E - Specificity, ^¶^PPV - Positive Predictive Value, ^**^NPV - Negative Predictive Value, ^††^PWH - Prince of Wales Hospital, ^‡‡^CITT - Cincinnati Individual Transfusion Trigger Emergency, ^§§^OR - Odds Ratio, ^||||^MTS - Massive Transfusion Score, ^¶¶^(P) - Prospective

**Figure 5:**
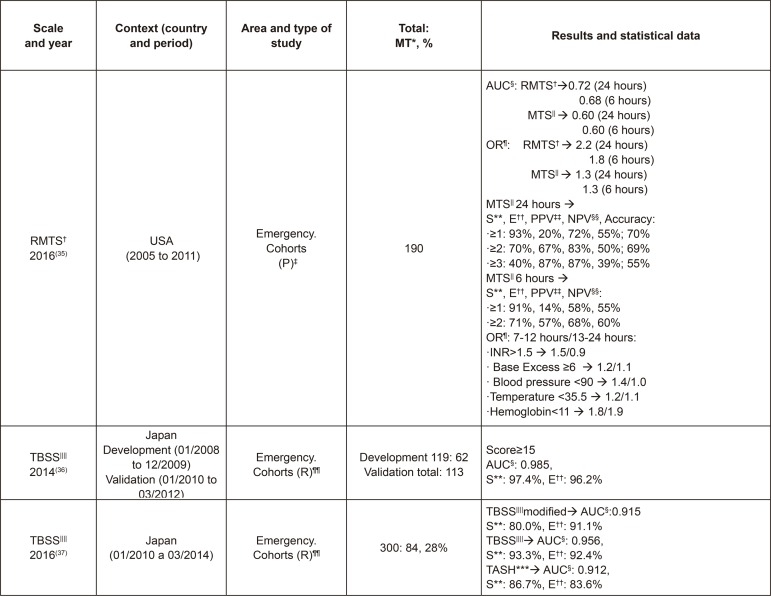
Characteristics of the studies on Revised Massive Transfusion Score (RMTS) and Traumatic Bleeding Severity Score (TBSS). Madrid, Spain, 2017

^*^MT - Massive Transfusion, ^†^RMTS - Revised Massive Transfusion Score, ^‡^(P) - Prospective, ^§^AUC - Area Under the Curve, ^||^MTS - Massive Transfusion Score, ^¶^OR - Odds Ratio, ^**^S - Sensitivity, ^††^E - Specificity, ^‡‡^PPV - Positive Predictive Value, ^§§^NPV - Negative Predictive Value, ^||||^TBSS - Traumatic Bleeding Severity Score, ^¶¶^(R) - Retrospective, ^***^TASH - Trauma Associated Severe Hemorrhage

In this way, the statistical data described in them were reviewed in order to compare the samples and their results, among other aspects, according to Figures 2, 3, 4 and 5.

As can be observed in Figures 2, 3, 4 and 5, of the 19 studies included that have led to the development scales for MT, most were conducted in the USA (8), followed by Germany (4) and Japan (2), and they were divided in two groups: those of retrospective nature (13) and those prospective in nature (6). In addition, except for three studies, their scope of analysis included the civilian population and they were performed in Emergency Hospitals (16), except for those carried out in Combat Hospitals (3). This characteristic directly influences the results, since the incidence of MT is altered. In these latter, higher percentages of MT are observed (between 26.5% and 44.5% among all traumatized patients), and lower percentages are observed in those conducted with a civilian population (between 2.12% and 28%).

### Statistical data from scales

Regarding the AUC, sensitivity, specificity, OR, NPV and PPV, the studies report different percentages associated with the cut-off points established in each scale, with a higher value corresponding to a higher prediction of MT. Considering the AUC as reference, the three scales with the highest values are TBSS^(^ ^36^ ^-^ ^37^ ^)^ (0.985 and 0.956), TASH^(^ ^25^ ^,^ ^37^ ^)^ (0.912 and 0.905) and ABC^(^ ^29^ ^)^ (0.906 at 6h and 0.903 at 24h). As for sensitivity, the TBSS^(^ ^36^ ^-^ ^37^ ^)^ shows higher values (97.4%, 93.3%) when compared to other scales, such as the TASH^(^ ^37^ ^)^ (86.7%) and the RMTS^(^ ^35^ ^)^ (91% at 6h and 93% at 24h). Regarding the specificity, higher values were achieved in the PWH^(^ ^5^ ^,^ ^32^ ^)^ (99.7% and 97.11%) and *Vandromme* ^(^ ^31^ ^)^ (98%), followed by the TASH^(^ ^24^ ^-^ ^25^ ^,^ ^37^ ^)^ (97%, 83.6%) and the TBSS^(^ ^36^ ^-^ ^37^ ^)^ (96.2%, 92.4%). In general, most of them describe high NPVs, thus avoiding the undertriage of patients, generally exceeding 90%, as in the cases of the *Vandromme* ^(^ ^31^ ^)^ (99%), PWH^(^ ^32^ ^)^ (96.6%) and ETS^(^ ^23^ ^)^ (98.4%). As can be seen in Figures 3, 4 and 5, in some studies, NPVs are associated with individual triggers, but not with the whole scale, as with the OR, and it is highlighted the values OR>7 and NPV>80%, with Hb≤11 g/dl^(^ ^26^ ^,^ ^30^ ^,^ ^34^ ^)^, FAST positive^(^ ^32^ ^-^ ^34^ ^)^, SBP≤90 mmHg^(^ ^32^ ^-^ ^34^ ^)^, INR>1.5^(^ ^33^ ^-^ ^34^ ^)^ and BE≥6^(^ ^33^ ^-^ ^34^ ^)^. Finally, the PPVs corresponding to a more accurate prediction of MT are the upper scores in the scales RMTS≥3 at 24 hours^(^ ^35^ ^)^ (87%), PWH≥6^(^ ^5^ ^)^ (70.59%), *McLaughlin* ^(^ ^27^ ^)^ (66.4%), and when there are two positive variables in *Larson* ^(^ ^30^ ^)^ (54%).

### Complementary studies

Furthermore, as mentioned above, in this scoping review 17 other articles were included to obtain a more detailed understanding of the study theme. Thus, other researches with comparative assessments between the scales for MT and the specific analyzes on the clinical and physiological predictive triggers were identified, which consisted of 10 original and 7 review studies.

By comparing it with a validation study of the RMTS^(^ ^35^ ^)^ that describes an AUC in the RMTS (0.68 at 6h and 0.72 at 24h) higher than that in the ABC (0.58 at 6h and 0.51 at 24h) of other studies^(^ ^38^ ^)^, it is observed that this latter scale is superior in terms of sensitivity (75%) and NPV (97%). Similarly, PWH is considered a useful scale with high specificity (99.7%) and PPV (82.9%), in comparison with the other the scales^(^ ^38^ ^)^. Moreover, after a revision of the literature, other acceptable AUCs of about 0.89 were described for the TASH^(^ ^20^ ^-^ ^21^ ^,^ ^39^ ^)^, PWH^(^ ^38^ ^-^ ^39^ ^)^ and ABC^(^ ^21^ ^)^. Finally, other scales such as *Schreiber* are highlighted for their sensitivity (85.8%), *Larson* for its specificity (80.4%) and TASH for its PPV (18.9%) and NPV (98.8%), in studies with other samples and settings^(^ ^39^ ^)^.

### Definition of Massive Transfusion

One aspect that must be highlighted is that, most of the studies included for the selection of patients for MT, consider the administration of ≥10 Units at 24 hours^(^ ^40^ ^-^ ^41^ ^)^, although other intervals are also analyzed in some of them for obtaining the sample. Among these thresholds is the *Critical Administration Threshold* (CAT) that is defined as the administration of ≥3 units at 1 hour^(^ ^21^ ^,^ ^42^ ^-^ ^43^ ^)^, and others, such as ≥4 units at 4 hours^(^ ^41^ ^,^ ^44^ ^)^, ≥5 units at 4 hours^(^ ^45^ ^)^ or at 6 hours^(^ ^29^ ^,^ ^34^ ^-^ ^35^ ^,^ ^46^ ^)^. Other tendencies have been found, which describe the patient as belonging to the MT group when there are blood requests without cross-matching, or blood group 0, as this has been identified as a good predictor for initiating the MT strategy^(^ ^35^ ^,^ ^38^ ^,^ ^41^ ^,^ ^44^ ^,^ ^47^ ^)^.

### Specific triggers

In addition to the research studies on the scales, other studies examine the triggers at the individual level. In a study carried out last year, the continuous monitoring of patient’s vital signs was carried out on their arrival at the hospital, through the measuring of HR and SBP, in order to associate them with MT prediction. It was concluded that at 10-15 minutes of their arrival these variables were significant in that field of study^(^ ^41^ ^)^. Another widely studied variable is Hb, whose scale range varies, being considered as a positive trigger when its values are lower than 11 g/dl^(^ ^24^ ^-^ ^26^ ^,^ ^30^ ^,^ ^33^ ^-^ ^35^ ^)^, although some more critical values, below 7, have been considered^(^ ^24^ ^-^ ^25^ ^,^ ^38^ ^)^, so there is not a specific value associated with a decrease in mortality, and it may oscillate between the two figures mentioned^(^ ^48^ ^)^.

Regarding the study on other triggers, temperature is usually not evaluated^(^ ^25^ ^,^ ^34^ ^)^ although it is considered as relevant in some studies^(^ ^33^ ^,^ ^35^ ^)^, and the INR has a high predictive value if ≥1.5^(^ ^26^ ^,^ ^33^ ^-^ ^35^ ^)^, as well as the presence of penetrating trauma mechanism and FAST positive^(^ ^22^ ^,^ ^32^ ^-^ ^37^ ^)^.

The new tendencies report Fibrinogen and BE^(^ ^46^ ^,^ ^49^ ^-^ ^50^ ^)^ as individual predictors of MT, which stand out because they diminish early, even before the other coagulation factors^(^ ^49^ ^)^. The prothrombin time (PT) and the activated partial thromboplastin time (aPTT)^(^ ^46^ ^,^ ^51^ ^)^, among others, used to determine Acute Traumatic Coagulopathy (ATC) are examined using the PROMMTT sample^(^ ^52^ ^)^ and its subsequent investigations^(^ ^34^ ^,^ ^46^ ^,^ ^53^ ^)^, which show their alteration but do not determine a fixed interval with regard their definition.

Finally, there is a clear need for a MT protocol or universal DCR^(^ ^41^ ^,^ ^54^ ^)^, since both the mortality of the traumatized patient and the need for blood units during hospitalization can be reduced through the unification of criteria and strategies of action.

## Discussion

The MT strategy has a low incidence in the total population, but its repercussions bring with it a large amount of material, personal and organizational health resources^(^ ^1^ ^,^ ^4^ ^,^ ^6^ ^,^ ^8^ ^,^ ^15^ ^-^ ^16^ ^,^ ^41^ ^)^. This incidence, described in the studies included in the review and expressed as percentage in relation to the traumatized population, will depend on the sphere where the sample was collected and the inclusion criteria used^(^ ^20^ ^-^ ^37^ ^)^. Despite this discrepancy, the receivers of MT do not usually exceed 15%^(^ ^28^ ^-^ ^29^ ^,^ ^31^ ^-^ ^32^ ^,^ ^38^ ^-^ ^39^ ^,^ ^41^ ^-^ ^44^ ^)^.

From these results, a particular and general analysis of all predictive scales for MT and their triggers is obtained, and it is possible to identify two subgroups of variables, the clinical and the analytical ones. Thus, those authors who only use clinical variables as triggers, regardless of the laboratory values^(^ ^20^ ^-^ ^22^ ^,^ ^24^ ^)^, justify their decision based on the need to perform MT early, and argue that an analysis of such variables would cause a delay in the administration of units because of their complex calculations^(^ ^32^ ^,^ ^40^ ^)^ and the use of non-immediate complementary tests^(^ ^36^ ^-^ ^37^ ^,^ ^40^ ^)^. However, when the results accuracy with the scales that combine the two types of triggers is taken into account, there is a significant improvement in the effectiveness of this decision, despite the later start of the strategy, as with the scales ABC, TASH and TBSS^(^ ^24^ ^-^ ^25^ ^,^ ^28^ ^-^ ^29^ ^,^ ^36^ ^-^ ^37^ ^)^.

Similarly, in spite of the high values described in the TASH and ABC, in terms of specificity, they have low sensitivity and lead to undertriage in many occasions. However, they are considered acceptable by some authors^(^ ^38^ ^,^ ^40^ ^)^ when they show high NPVs, because if such a situation occur and the protocol is activated and, ultimately, it is no longer necessary, there would be the possibility of returning the requested blood products back to the Blood Bank.

As regards to the triggers investigated at the individual level, Hemoglobin is part of most studies, but there is no consensus on its critical range for activating the MT protocol^(^ ^24^ ^-^ ^26^ ^,^ ^30^ ^,^ ^32^ ^-^ ^35^ ^,^ ^38^ ^-^ ^39^ ^,^ ^48^ ^)^. A possible explanation is that it varies depending on the moment in which the analytical value is obtained, influencing both the time elapsed since the incident and the strategies performed before obtaining the first sample^(^ ^48^ ^)^.

Regarding the concept of massive transfusion, a variation in its definition has been observed, which it is coincident with the chronological progression over the years. Thus, in more recent studies, MT triggers are usually analyzed more frequently in the early hours, between one and four hours, since the critical level of the individual is higher at that period^(^ ^21^ ^,^ ^41^ ^-^ ^44^ ^)^.

As a main limitation found, it can be highlighted that in many studies the need for MT itself is not described, but the use of this strategy^(^ ^35^ ^,^ ^41^ ^)^. That is, it is difficult to differentiate between those who really need MT and those who receive it. Similarly, the samples and environments used in the studies are not equivalent or easily comparable, except for those that are carried out by the same research group or arise from the same selection of patients as, for example, in the MTS^(^ ^34^ ^-^ ^35^ ^,^ ^52^ ^)^.

Finally, the need to use scales arises from the presence of atypical or not apparent hemorrhages, since there is no doubt on how to proceed when there are external bleeds. In addition to all this, the fact that not all health personnel are specialized in the care for the traumatized patient would lead to a disparity of criteria, since there is in the same Hospital Emergency a large number of professionals involved in their care, with different profiles and types of residences^(^ ^41^ ^)^. Therefore, with the implementation or use of the same scale, both the team’s work and the quality of care provided could be facilitated with the application of the same protocol.

## Conclusion

The variability of universal criteria regarding the massive transfusion triggers in traumatized patients has led to the creation of different scales. Therefore, the validation studies of these scales are relevant to reach an agreement about the criteria on when to initiate this strategy.

Therefore, the conclusions of this *scoping review*, based on the characteristics of the selected studies, can be summarized as they are quantitative, predominantly retrospective in nature and focused on a single care field: the emergency hospital. This arises the need to propose the development of new research studies, in which the different scales and the massive transfusion triggers are analyzed in the two initial critical care areas for the subjects described, the hospital emergency and the out-of-hospital emergency, using the same sample.

However, this review is considered useful not only in the research field, but also in the care field, since it compares the existing scales for massive transfusion and their main conclusions, aiming at reaching a common protocol of action for urgency and emergency health personnel. Thus, by establishing this continuity, it would be possible to follow the massive transfusion triggers and the pertinence of initiating this strategy, identifying the areas for potential improvement, and proposing a further formation on massive transfusion and severely traumatized patients.
